# Collapsing Glomerulopathy in Identical Twins With Lupus and High-Risk Apolipoprotein L1 (*APOL1*) Genotype

**DOI:** 10.1016/j.ekir.2021.06.005

**Published:** 2021-06-19

**Authors:** Margaret DeOliveira, Colby Feeney, Caroline Leahy, Sarah Nystrom, David N. Howell, Samira S. Farouk, Ming Wu, Opeyemi A. Olabisi, Matthew A. Sparks

**Affiliations:** 1 Duke University School of Medicine, Durham, North Carolina, USA; 2 Department of Medicine and Pediatrics, Duke University School of Medicine, Durham, North Carolina, USA; 3 Department of Pathology, Duke University School of Medicine, Durham, North Carolina, USA; 4 Division of Nephrology, Department of Medicine, Icahn School of Medicine at Mount Sinai, New York, NY, USA; 5 Division of Nephrology, Department of Medicine, Duke University School of Medicine, Durham, North Carolina, USA; 6 Renal Section, Durham VA Health Care System, Durham, North Carolina, USA; 7 Department of Pathology, NYU Langone Medical Center, NY, New York, USA

## Introduction

Collapsing glomerulopathy (CG) is characterized by global or segmental collapse of the glomerular capillary tuft along with proliferation of overlying parietal epithelial cells. It is associated with multiple etiologies including human immunodeficiency virus (HIV), severe acute respiratory syndrome coronavirus-2 (SARS-CoV-2), autoimmune diseases including systemic lupus erythematosus (SLE), malignancy, drug exposures, and other viral illnesses.^1^ Patients usually present acutely with severe acute kidney injury (AKI) and proteinuria. Irrespective of the etiology, CG is a marker of poor prognosis. It is predominantly seen in individuals of recent West African ancestry. Cumulative evidence demonstrates that carriage of 2 apolipoprotein L1 (*APOL1*) kidney risk variants (KRVs) increases the risk of CG in SLE,^2^ HIV,^3^ transplanted donor kidneys,^4^^,^^5^ and SARS-CoV-2.^6^ However, not all carriers of 2 *APOL1* KRVs develop CG. Attempts to explain this apparent incomplete penetrance gave rise to the “2-hit hypothesis” that proposes that a second hit—either environmental trigger or additional genetic factors other than 2 *APOL1* KRVs—is necessary for pathogenesis of *APOL1*-associated CG. Because CG is associated with diseases with high inflammatory cytokines, and as iatrogenic interferons have been associated with CG,^7^, ^8^, ^9^ it has been speculated that environmental factors or conditions such as SLE, which raise levels of circulating interferons, would constitute second hits. However, combination of 2 APOL1 KRVs and known second hits such as HIV or SLE results in APOL1-associated kidney disease (AAKD) in only a fraction of all cases, raising the possibility that the additional genetic or epigenetic modifiers are essential for onset or progression of AAKD. Genome-wide association studies of heterogenous patient population did not reveal reproducible common genetic modifier of AAKD.S1 The failure of these genome-wide association studies is likely driven by multiple factors including the possibility of insufficient power to detect polygenic risk modifiers with modest effect sizes in heterogenous population. Here, we present 2 cases of identical twins with a history of SLE who developed APOL1-associated CG. We propose that this twin-case adds evidence in support of the possible role of a patient’s genetic background in the pathogenesis of APOL1-associated CG and that the identical genome of monozygotic twins with APOL1-associated CG may be ideal for discovering such genetic modifiers of AAKD.

## Case Reports

### Twin A

**Figure 1:**
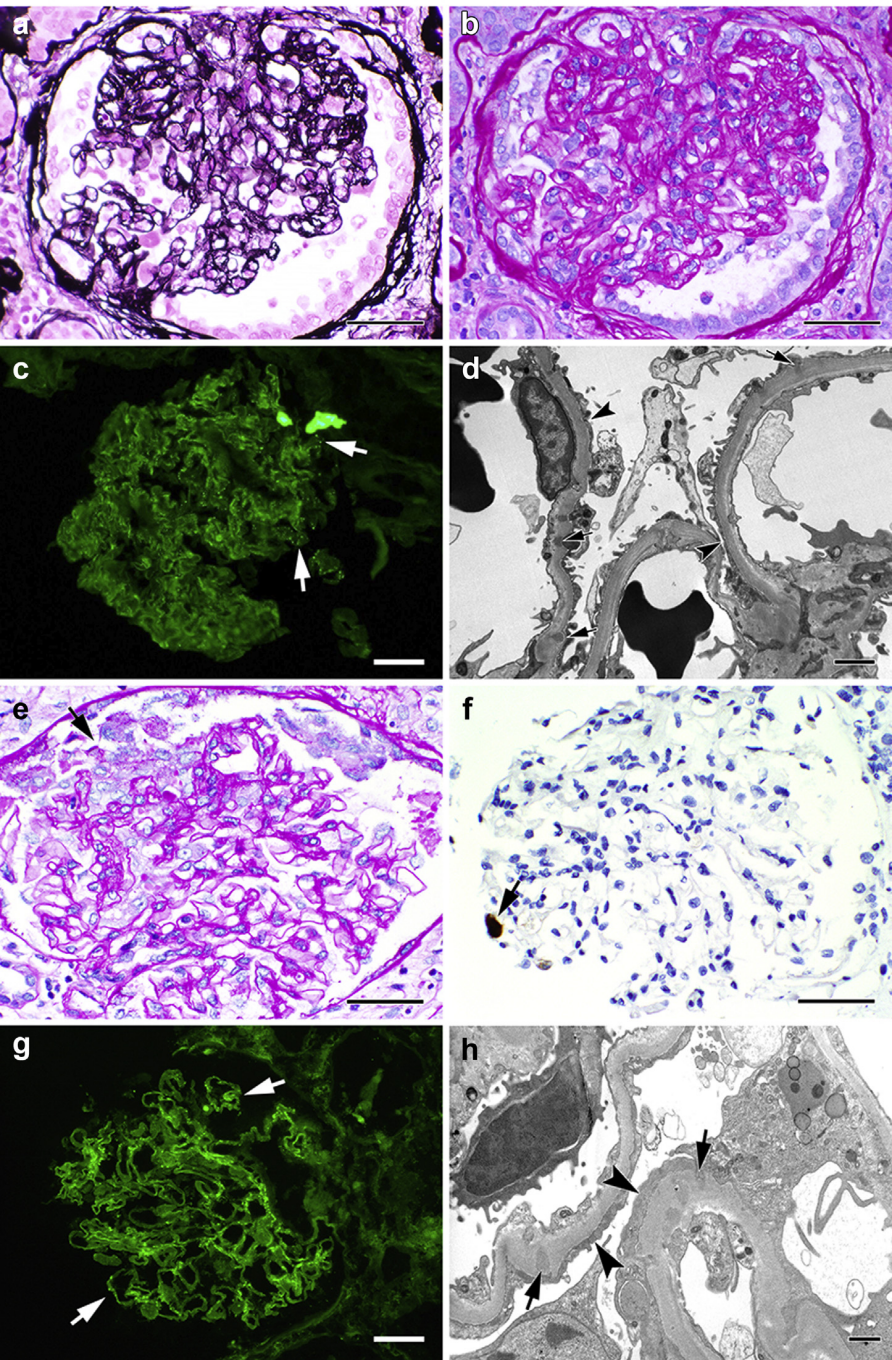
Kidney biopsy findings. (a-d) Twin A. (a) Glomerulus with global collapse seen on Jones silver stain (bar = 50 μm). (b) Same glomerulus seen on periodic acid–Schiff stain (bar = 50 μm). (c) Immunofluorescent stain for IgG, showing sparse, punctate staining of glomerular capillary loops (arrows) (bar = 50 μm). (d) Electron micrograph of portion of a glomerulus, showing rare subepithelial and intramembranous immune complex deposits (arrows) and patchy podocyte foot process effacement (arrowheads) (bar = 2 μm). (e-h) Twin B. (e) Glomerulus with area of segmental collapse and podocyte reactive change (arrow) (periodic acid–Schiff stain, bar = 50 μm). (f) Immunohistochemical stain for cytomegalovirus showing single immunoreactive glomerular cell (arrow) (bar = 50 μm). (g) Immunofluorescent stain for IgG, showing sparse, punctate staining of glomerular capillary loops (arrows) (bar = 50 μm). (h) Electron micrograph of portion of a glomerulus, showing rare subepithelial and intramembranous immune complex deposits (arrows) and diffuse podocyte foot process effacement (arrowheads) (bar = 1 μm).

**Figure 2:**
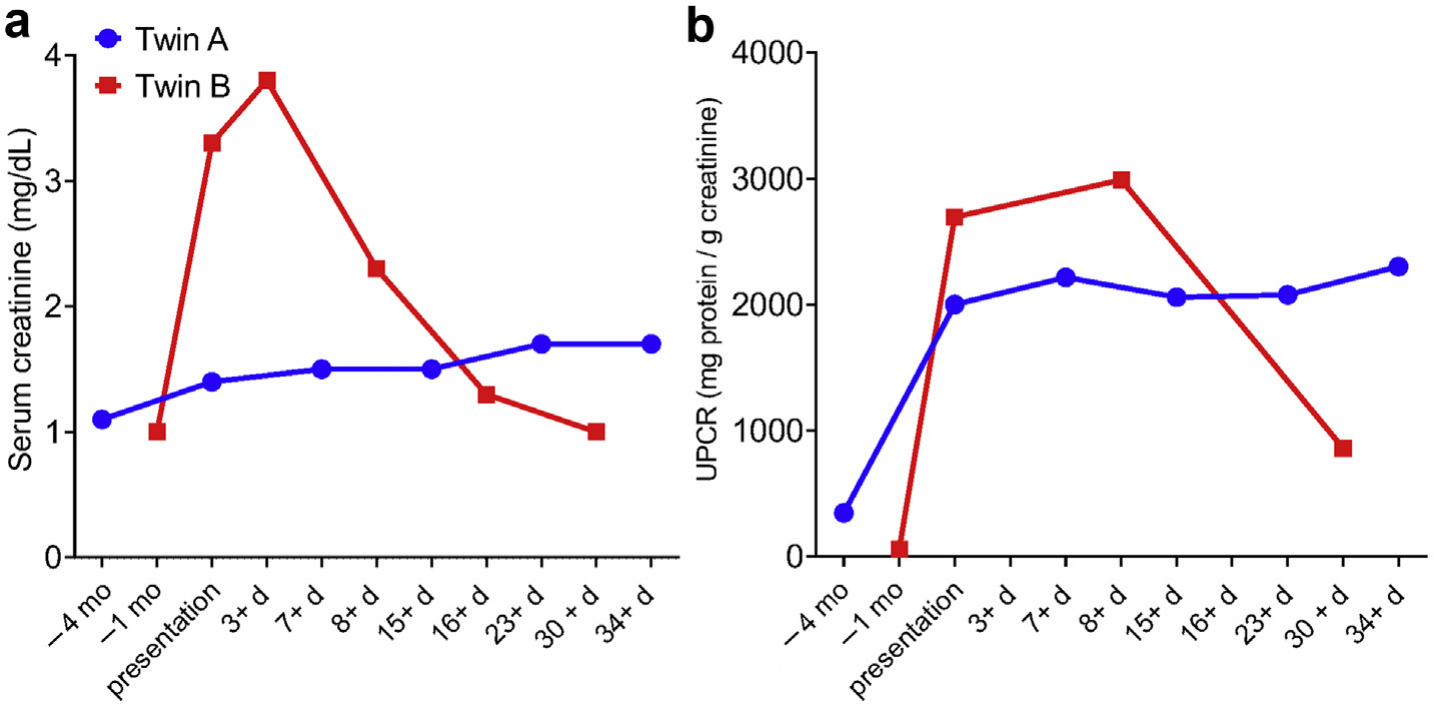
Trend of serum urine protein creatinine ratio (UPCR) and serum creatinine for each twin before presentation, at presentation, and after initiation of their respective treatments.

A 23-year-old man, with a history of class IV lupus nephritis at age 10, presented with AKI and persistent proteinuria (about 750 mg/g) in the summer of 2019. He was noted to have increasing edema in bilateral lower extremities and hand for several weeks. He had no infectious symptoms at the time, including congestion, cough, rhinorrhea, joint pains, or rashes. His examination was notable for body temperature of 36.8 °C, blood pressure 118/60 mm Hg, heart rate 61 beats per a minute, and bilateral 1+ pitting edema in the legs. He did not have rashes, joint swelling, or abdominal tenderness. Laboratory data revealed proteinuria and AKI. Urinalysis with trace blood, large protein, 1 red blood cell, white blood cell count 4800/L, hemoglobin 12.5 g/dl, platelet count 228,000/L, C3 107 mg/dl, C4 20 mg/dl, anti–double-stranded DNA 4 IU/ml, erythrocyte sedimentation rate 7 mm/h, C-reactive protein 0.2 mg/l. Other labs were unremarkable. He underwent a kidney biopsy that showed CG without tubuloreticular inclusions but accompanied by scattered capillary loop immune complex deposits (Figure 1a to d). He was started on prednisone 60 mg daily and continued on hydroxychloroquine 200 mg twice daily, losartan 50 mg daily, and mycophenolate 500 mg twice daily. HIV antigen/antibody was not detected. His creatinine and proteinuria did not improve with treatment, as illustrated in Figure 2. *APOL1* genotyping revealed G1/G2 about 18 months after his initial diagnosis of CG.

### Twin B

A 24-year-old man with SLE, diagnosed 8 years ago, presented with periumbilical abdominal pain, nonbloody diarrhea, and dark-colored urine for 3 weeks in the summer of 2020. He has no prior history of lupus nephritis. His current medications included hydroxychloroquine 400 mg daily, mycophenolate mofetil 1500 mg every morning and 1000 mg every night, and prednisone 5 mg daily. Soon after presentation, he was admitted to the hospital with AKI and proteinuria (urine protein-to-creatinine ratio was 2695 mg/g) (see Figure 2 for trend).

On admission, body temperature was 37.3 °C, blood pressure 141/88 mm Hg, and heart rate 87 beats per minute; there was tenderness to palpation in the left lower quadrant without rebound or guarding and no oral ulcers, rash, joint abnormalities, or peripheral edema. Laboratory findings on admission were as follows: white blood cell count of 5900/L, hemoglobin 13.6 g/dl, platelets 203,000/L, sodium 129 mmol/l, potassium 3.8 mmol/l, bicarbonate 23 mmol/l, blood urea nitrogen 33 mg/dl, creatinine 3.3 mg/dl, albumin 2.5 g/dl, aspartate transaminase 81 U/L, alanine transaminase 117 U/L, erythrocyte sedimentation rate of 28 mm/h, C-reactive protein 1.46 mg/dl, and HIV-1/HIV-2 antibodies were not detected. Serum cytomegalovirus polymerase chain reaction was 35,536 IU/ml, Epstein-Barr virus serum polymerase chain reaction 5235 IU/ml, anti–double-stranded DNA 71 IU/ml, and COVID-19 polymerase chain reaction negative. On the ultrasonogram, kidneys appeared normal size with diffusely increased cortical echogenicity bilaterally and no hydronephrosis.

As AKI persisted with creatinine of 3.8 mg/dl, despite resolution of diarrhea, a kidney biopsy was performed, which revealed CG without tubuloreticular inclusions but accompanied by scattered capillary loop immune complex deposits. Immunohistochemical staining for cytomegalovirus revealed a single immunoreactive glomerular cell (Figure 1e to h)

Valganciclovir treatment was initiated and cytomegalovirus viral load was undetectable after 1 month. Urine protein-to-creatinine ratio and creatinine improved to 862 mg/g and 1 mg/dl, respectively, 1 month after presentation as illustrated in Figure 2. *APOL1* genotyping revealed G1/G2. The twin brothers shared a common environment for the first 18 years and lived apart afterward.

## Discussion

A key unanswered question about AAKD is why only 15% to 20% of carriers of 2 KRVs develop AAKD in their lifetime whereas the remaining 80% remain free of kidney disease.S2 Need for second hits—environmental trigger or additional genetic factors—was proposed to explain this apparent incomplete penetrance. Genome-wide association studies have thus far been unsuccessful at revealing common and specific genetic modifiers of AAKD.S1 Therefore, environmental triggers have expanded in the collective imagination as the sole second hits. The current twin-case report adds to the evidence that supports the possibility that unidentified genetic determinants, in addition to known environmental triggers, contribute to the pathogenesis of APOL1-associated CG.

There are recent case reports of transplant donor APOL1-associated focal segmental glomerulosclerosis with and without CG independent of recipients APOL1 genotypeS3^,^S4 and twin donor-recipient APOL1-associated focal segmental glomerulosclerosis.S3 To our knowledge, this is the first case report of biopsy-confirmed, APOL1-associated CG in identical twins with SLE. Despite sharing an identical genome and identical environments until age 18 years, the course of SLE was different for each twin. Although twin A developed LN at age 10 years, twin B’s SLE was not diagnosed until age 16 years, and he did not develop kidney complications before presentation. These divergent disease courses underscore the stochastic nature of SLE. Importantly, regardless of their unique SLE courses, both twins ultimately arrived at the same renal histopathologic terminus of CG, highlighting the strong contribution of their shared genetic background.

Aspects of this twin-case are similar to aspects of many of the cited prior case reports. For instance, as true for twin B, systemic viral infection (cytomegalovirus or Epstein-Barr virus) was the presumed second-hit trigger of CG in many of the prior cases. However, like twin A, no viral infections or other known second hits were identified in many of the prior cases of APOL1-associated CG also. We believe that these exceptions indeed prove the rule. If the combination of viral infection plus 2 KRVs or SLE plus 2 KRVs are sufficient to trigger APOL1-associated CG, then the frequency of CG should be higher than observed when these 2 hits are present. The fact that known environmental second hits are not always present in cases of APOL1-associated CG and that the combination of viral infections or SLE and KRVs results in APOL1-associated CG only in some but not all cases strongly suggest that other modifiers contribute to pathogenesis of APOL1-associated CG. Moreover, enrichment of APOL1-associated CG among kidneys that share identical genomes suggests that the elusive “other modifiers” of APOL1-associated CG are likely genetic in nature. The likelihood of discovering these genetic modifiers could increase if future genome-wide association studies focus on genomes of identical twins with APOL1-associated CG. In conclusion, these 2 cases highlight the need for *APOL1* testing in patients of recent West African descent with SLE and CG, even when other possible etiologies are identified (Table 1).

**Table 1:** Teaching points

• Carriers of 2 risk alleles of *APOL1* have a high risk of developing a spectrum of glomerular disorders, including lupus collapsing glomerulopathy. A low threshold for genotyping appropriate patients of recent West African ancestry will aid timely diagnosis.
• The incomplete penetrance of kidney disease risk of high-risk *APOL1* genotype suggests contribution of genetic or epigenetic modifiers.

## Disclosure

OAO is supported by DP2DK124891. All the other authors declared no competing interests.

## Patient Consent

Written consent was obtained from both brothers for this case report.

## Supplementary Material

Supplementary File (PDF)

**Supplementary References.**
