# Supplementary material for: Collapsing Glomerulopathy in Identical Twins With Lupus and High-Risk Apolipoprotein L1 (APOL1) Genotype
Source: Kidney Int Rep. 2021 Jun 19;6(9):2501–4. doi: 10.1016/j.ekir.2021.06.005 (PMC8418970; doi:10.1016/j.ekir.2021.06.005)
Supplement: Supplementary File (PDF) [file mmc1.pdf]

S1. Langefeld CD, Comeau ME, Ng MCY, et al. Genome-wide association studies suggest that APOL1-environment interactions more likely trigger kidney disease in African Americans with nondiabetic nephropathy than strong APOL1-second gene interactions. *Kidney Int.* 2018;94(3):599-607.

S2.Dummer PD, Limou S, Rosenberg AZ, et al. APOL1 Kidney Disease Risk Variants: An Evolving Landscape. *Semin Nephrol.* 2015;35(3):222-236.

S3.Kofman T, Audard V, Narjoz C, et al. APOL1 polymorphisms and development of CKD in an identical twin donor and recipient pair. *Am J Kidney Dis.* 2014;63(5):816-819.

S4. Chang JH, Husain SA, Santoriello D, et al. Donor's APOL1 Risk Genotype and "Second Hits" Associated With De Novo Collapsing Glomerulopathy in Deceased Donor Kidney Transplant Recipients: A Report of 5 Cases. *Am J Kidney Dis.* 2019;73(1):134-139.
